# The Influence of Alkali-Free Shotcrete Accelerators on Early Age Hydration and Property Development within Cement Systems

**DOI:** 10.3390/ma15196907

**Published:** 2022-10-05

**Authors:** Wei Wang, Luping Zeng, Shuang Du, Min Qiao, Junsong Chen, Bosong Zhu

**Affiliations:** 1 Jiangsu Key Laboratory of Construction Materials, School of Material Science and Engineering, Southeast University, Nanjing 211189, China; 2 State Key Laboratory of High Performance Civil Engineering Materials, Jiangsu Sobute New Materials Co., Ltd., Nanjing 211103, China

**Keywords:** alkali-free accelerator, early age hydration, calcium fluoride, ettringite

## Abstract

Fluoride-containing alkali-free setting accelerators are a common type of admixture used in tunnel shotcrete but few studies in the literature focus on the effect of their fluoride compounds on the setting and hardening properties of accelerated cement paste under low environment temperatures. Tunnel shotcrete in cold regions or winter construction periods would be obviously influenced by low environment temperatures, especially for its fast setting and quick support applications. The objective of this work is to evaluate the early age hydration behavior of different accelerated cement pastes under 20 °C and 5 °C environment temperatures. In this study, setting time measurement, early age strength development, hydration ion leaching concentration, isothermal calorimetry, X-ray diffraction, and ESEM were performed on cement systems prepared with a non-fluoride alkali-free accelerator (aluminum sulfate solution with over 60% solid content) and a designed fluoride-containing alkali-free setting accelerator (aluminum sulfate and fluoride compound). The results showed that the fluorides obtained in alkali-free accelerators promote C3S dissolution and massive ettringite needles together with monosulfoaluminate (AFm) hydrate formation, thus leading to a quicker setting effect and low sensitivity to low environment temperatures than in non- fluoride groups. However, the rate of mechanical strength development of cement pastes hydrated within 24 h was decreased obviously when fluorine-containing alkali-free accelerator was used. This phenomenon is mainly related to the crystallization of thin-plate shape calcium fluoride (CaF_2_) formations and promoted conversion of ettringite to monosulfoaluminate hydrate in the accelerating period, thus weakening the denseness of C-S-H gel and inhibiting alite further hydration.

## 1. Introduction

Sprayed concrete is a special kind of cement-based material characterized by exclusive construction equipment and a specific placement method, defined as ‘‘concrete pneumatically projected onto a surface at high velocity” [1,2]. High-pressured air is usually used to provide the transport power and enable the shot concrete to be attached and consolidated on the tunnel rock, 3D-print concrete, or old constructions [3,4]. However, in winter or high-altitude areas, the efficiency of shotcrete construction decreases, usually due to its slower hydration rate. Some winter construction measures are published to relieve the low hydration reaction problems, such as heating aggregates or mixing water [5]. To some extent, proper alkali-free accelerators with low-temperature sensibility are still very useful for enhancing cement hydration at low temperatures.

Liquid setting accelerators are often used in shotcrete and could reach 4–12% mass content of pure cement, thereby accelerating the coagulation and hardening rate of cementitious matrices. In some cases, the addition of setting accelerators enables sprayed concrete for vertical and overhead applications in tunnel excavating and reduces the rebound concrete loss [6,7]. In general, the early age hydration of accelerated cement systems could be significantly affected by the abundant chemical components obtained in setting accelerators [8,9]. Setting accelerators are mainly divided into alkaline groups and alkaline-free groups according to the alkaline metal content, calculated as equivalent sodium oxide content [10,11]. Traditional alkaline liquid setting accelerators constituting sodium silicate or sodium aluminate present a safety threat to operators in the process of construction and damage the strength development of shotcrete in the application. Therefore, this promotes the use of alkali-free setting accelerators in tunnel construction, especially since the requirements of support performance of the initial liner become increasingly higher in the sophisticated surrounding rock and severe environments [12].

An alkali-free accelerator containing fluoroaluminate is one kind of setting accelerator involving the combination of aluminum salts and fluoride salts or fluoride acid [13]. Due to its high acidic fluoride content, fluoride-containing alkali-free setting accelerators have a favorable compatibility with cement, hence enabling relatively shorter setting times. However, the addition of fluoride-containing alkali-free setting accelerators might arouse some safety issues for workers and iron-made equipment erosion problems. Furthermore, much lower mechanical strength at the hydration of 6 h or 24 h is another application problem for tunnel support since they are reported to accelerate C_3_A hydration but retard C_3_S hydration due to the coating effect of CaF_2_ and F-Ca-Al compounds [14]. Large-scale construction of railways and highway tunnels in mountainous areas might encounter deformation of soft rock or hard rock burst, hence expecting the higher early age strength development of shotcrete. Only high concentration alkali-free accelerators without fluoroaluminate are able to reach the 6 h or 8 h compressive strength index of 1 Mpa of mortar, conforming to the enterprise standard of China National Railway Group QCR 807-2020, thus leading to enough early age strength of shotcrete [15]. Despite that, fluoride-containing alkali-free setting accelerators are still widely used in several tunnel constructions due to their better accelerating effect and lower cost.

Although some works have been reported about fluoride-containing alkali-free setting accelerators and shotcrete application in cold areas, more focus is on frost damage and other durability properties [5,13,14]. It is still essential to evaluate the early age hydration behavior of different accelerated cement pastes under low environment temperatures, compared to room temperatures. Moreover, the effect of fluoride-containing alkali-free setting accelerators on hydration leaching ion characteristics and hours of compressive strength have not been reported. Therefore, this study on the subject continues to optimize the environment temperatures and accelerating materials.

Therefore, the main objective of this study is to examine the early age hydration behavior of different accelerated cement pastes under low environment temperatures by means of setting time, compressive strength, hydration dissolution ions, heat evolution, X-ray diffraction analysis (XRD), scanning electron microscope (SEM), and other test methods. In order to achieve the proposed objective, two kinds of alkali-free accelerators with or without fluoride compounds were designed and synthesized. Furthermore, different accelerated cement paste specimens were prepared and cured under 20 °C and 5 °C environment temperatures. The results obtained in this study promote the development of non-fluoride alkali-free accelerators and provide the reasons for the early strength problems of shotcrete in a low-temperature environment.

## 2. Materials and Methods

### 2.1. Materials

Reference cement conforming with Chinese standard GB 8076-2008 was used in this study [16]. Table 1 presents the chemical and mineral compositions of cement. The density and Blaine fineness of the Portland cement were 3.08 g/cm^3^ and 356 m^2^/kg, respectively. Natural river sand confirmed with Chinese standard GSB 08-1337 was applied in this research. Two kinds of alkali-free accelerators (AFA) with and without fluoride were synthesized to analyze the hydration and strength evolution. In this study, the main components of alkali-free accelerators (AFA) were aluminate sulfate octadecahydrate (Al_2_(SO_4_)_3_ 18H_2_O) and ethanolamine (DEA or TEA).

### 2.2. Synthesis of AFA Accelerator

Alkali-free accelerator with fluoride (AFA-F) was prepared as follows. At first, 40 g magnesium fluorosilicate was dissolved in 360 g water in a laboratory flask for 25 min in a 60 °C water bath environment with a stirring speed of 200 r/min. Then, 500 g aluminum sulfate octadecahydrate (Al_2_(SO_4_)_3_ 18H_2_O) and 100 g diethanolamine (DEA) were added to the solution being kept warm and stirred for 1 h. Finally, the AFA-F was successfully synthesized.

Alkali-free accelerator without fluoride (AFA-H) was prepared as follows. First, 60 g di-ethanolamine (DEA) and 20 g triethanolamine (TEA) were dissolved in 275 g water. Then, 630 g aluminum sulfate octadecahydrate (Al_2_(SO_4_)_3_ 18H_2_O) was added into the solution with a high dispersion process for 2 h. Lastly, 10 g sepiolite, organically modified, and 5 g glycerinum were mixed into the solution above for half an hour. During the synthesis, the temperature was kept around 80 °C and the stirring speed was maintained at a linear velocity of 15 m/s. We lowered the temperature to 40 °C before discharging the material. The high-performance alkali-free accelerator was finally prepared. The physical properties and chemical compositions of AFA-F and AFA-H were listed in Table 2.

### 2.3. Mix Proportion of Cement Paste and Mortar

The mix proportions of cement paste and mortar used in the study were shown in Table 3. The AFA-F and AFA-H alkali-free accelerators were introduced at a dosage of 8.0% by cement weight (% bcw). Water cement (w/c) ratio in the cement paste and mortar were chosen as 0.35 and 0.50 separately and the water content of setting accelerators was deducted to assure the constant w/c ratio.

### 2.4. Experimental Methods

#### 2.4.1. Setting Time of Cement Paste and Compressive Strength of Mortar

The preparation steps, setting time, and compressive strength test methods of the accelerated cement pastes and all mortar specimens all followed the requirements of Chinese standard GB/T 35159-2017 [9]. However, there are two types of environments; besides the normal 20 °C environment condition, 5 °C was adopted by placing all experimental materials in that environment before testing. Specifically, setting time measured with the reference paste was conducted following another Chinese standard, GB/T 1346-2011, in order to figure out the effect of low environment temperatures on the setting and hardening characteristics of pure cement paste.

#### 2.4.2. Isothermal Calorimeter Test

In order to analyze the influence of AFA with and without fluoride on the early hydration evolution of cement paste, isothermal calorimetric tests were carried out. The calorimetric curves of hydrating cement paste with AFA were measured by TAM air micro-calorimeter (TAM 08, produced by TA company in New Castle, DE, USA). The cement paste was prepared with a water/cement ratio of 0.40. The setting accelerators were added immediately after mixing water and stirred slowly for 10 s. Then, 42.0 g of the reacted cement paste was placed in the sample cell and the exothermic rates and exothermic heats of hydration from 0 to 72 h at room temperature of 20 °C were recorded. The doses of setting accelerator AFA-F and AFA-H were 8% (% bcw) in the test.

#### 2.4.3. Hydration Ion Leaching Concentration

Hydration ion leaching concentration test was carried out in the cement leaching suspensions with a w/c ratio equal to 1.0 and the water content of setting accelerators was also deducted. Before the equipment test, the leaching suspensions were diluted by 100 times with deionized water and then filtrated with double filter paper (bore diameter, 30 μm) in order to remove possible insoluble impurities and unhydrated cement particles. Finally, the treated suspensions were determined by an inductively coupled plasma spectrometer (FMS 26, produced by Spectro company in Kleve, Germany). In particular, the fluoride ion concentration of cement suspension containing AFA-F was tested by ion chromatography (TOSOH IC 2010, produced by Dongcao chemical technology company in Shanghai, China).

#### 2.4.4. X-ray Diffraction

The mineralogical compositions of the accelerated cement paste were determined by the X-ray diffraction method (Advance D8, produced by Bruker company in Bremen, Germany). The X-ray patterns of cement pastes were collected in the 5–70° 2θ range with a step size of 0.02° 2θ, operating at 40 kV and 40 mA with a Cu-Ka source. Then, measured patterns were analyzed semi-quantitatively by Rietveld methods using Total Pattern Analysis Solutions software. The XRD samples were taken from the cement paste at the corresponding age, placed in anhydrous ethanol for 3~5 days to terminate the hydration process, then dried in a vacuum drying oven at 45 °C for 5~7 days grounded finely through a 0.045 mm square-hole sieve.

#### 2.4.5. SEM Analysis

Microstructural characterization of cement pastes was conducted by scanning electron microscopy (SEM), using an FEI QUANTA 250 microscope (QUANTA 250, produced by FEI company in Hillsboro, OR, USA) at an acceleration voltage of 15 kV and a 9.5–10.0 mm working distance. SEM images were obtained from cracked sections coated with a thin gold layer. Characteristic X-rays were collected by the energy dispersive spectrum analysis (EDS) for element composition analysis of hydration products.

## 3. Results and Discussion

### 3.1. Isothermal Calorimeter Analysis

**Figure 1:**
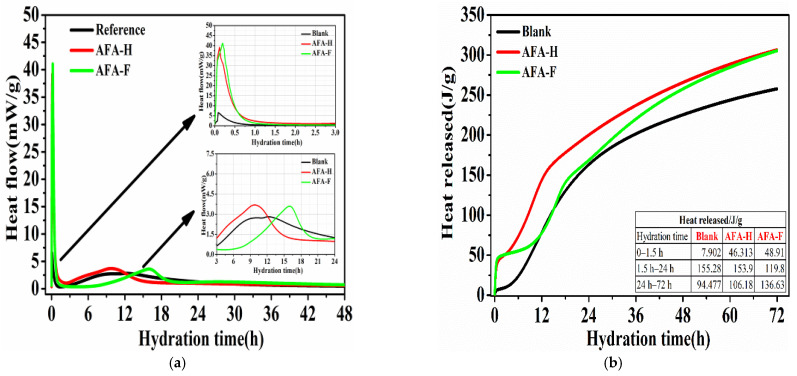
Heat flow (**a**) and heat released (**b**) curves of the cement paste containing liquid alkali-free accelerators with and without fluoride during the first 72 h of hydration at room temperature.

Heat flow and heat released curves of hydration until 72 h are shown in Figure 1. In the reference cement paste, the initial dissolution period lasts dozens of minutes or even one hour and abundant ions are released initially in this period due to the dissolution of cement minerals and initial hydration reaction of C_3_A phase [17], inducing the first exothermic peak with 6.62 mW/g. Then, three periods including induction period, accelerating period, and deceleration period happen orderly [18]. The second exothermic peak with 2.80 mW/g could be observed in the accelerating period at 12.3 h of hydration.

Compared with the reference paste, induction periods in accelerated pastes are shortened obviously and the initial period and accelerating period are also influenced remarkably with the use of alkali-free accelerators. In general, the first exothermic peak in the initial dissolution period reaches 39.2 mW/g for AFA-H and 41.1 mW/g for AFA-F, over 5–6 times than that measured for the reference paste. This higher heat flow observed in this period is generated by the fast formation of ettringite phases or other hydration products involved the C_3_A phase [14]. Additionally, a slight increase of the first exothermic peak in the cement paste containing alkali-free accelerators with fluoride may be noticed, compared with that without fluoride. This phenomenon is possibly related to the enhanced acidity of alkali-free accelerators when fluoride is used, thus promoting the dissolution process of alite phase and higher dissolution heat released [7]. On the other hand, the second exothermic peak in the accelerating period of the accelerated cement paste varies with each alkali-free accelerator. In the paste with AFA-H, an advanced and higher second exothermic peak may be observed at 9.7 h of hydration, corresponding to 3.69 mW/g of the heat flow. However, the second exothermic peak of paste with AFA-F is delayed to 16.1 h of hydration, while the reference one reaches 12.3 h of hydration. The delayed heat flow observed in this process implies the slower strength growth of cement mortar or shotcrete with the use of alkali-free accelerators containing fluoride.

Figure 1b mainly presents the calculated heat release of cement with each alkali-free accelerator. For the initial dissolution period from 0 to 90 min, much higher heat release of accelerated cement paste is observed and related to the fast formation of ettringite phases. However, for the hydration time from 90 min to 24 h, less heat release of cement paste containing AFA-F can be noticed compared to that with AFA-H and the reference one, also supporting the delayed accelerating period and possible lower initial mechanical strength development [14].

### 3.2. Hydration Ion Leaching Concentration

The dissolution process of gypsum phase and cement minerals like C_3_S or C_3_A phase in Portland cement can be reflected by ion concentration evolution in early age hydration. High aluminum ions introduced by alkali-free accelerators incorporated into cement minerals will lead to a fast and highly exothermic ettringite formation, thus affecting the early age hydration process and shortening the setting time of cement paste [19].

**Figure 2:**
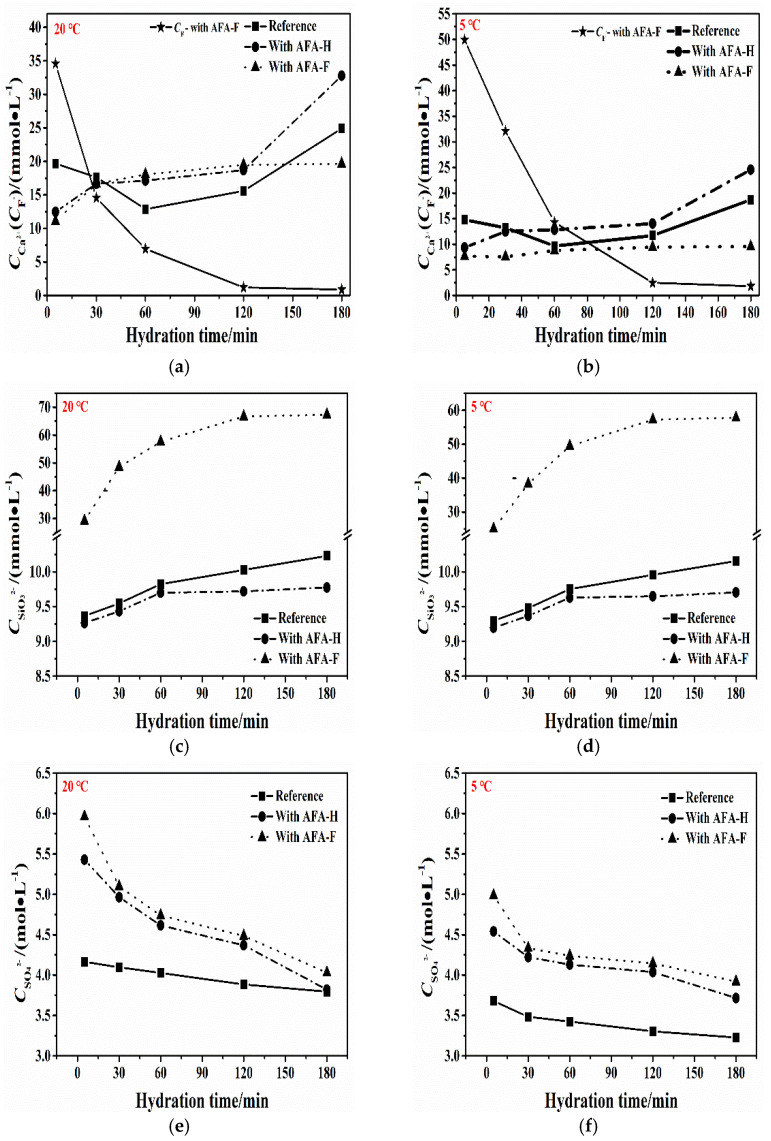
Hydration leaching ion concentration curves of the cement paste containing liquid alkali-free accelerators with and without fluoride during the first 180 min of hydration under 20 °C or 5 °C; (**a**) leaching concentration of Ca^2+^/F^−^ at 20 °C, (**b**) leaching concentration of Ca^2+^/F^−^ at 5 °C, (**c**) leaching concentration of SiO_3_^2^^−^ at 20 °C, (**d**) leaching concentration of SiO_3_^2^^−^ at 5 °C, (**e**) leaching concentration of SO_4_^2^^−^ at 20 °C, (**f**) leaching concentration of SO_4_^2^^−^ at 5 °C.

Figure 2 shows the evolution of the dissolved ions concentration of hydrated Portland cement with and without alkali-free setting accelerators at the beginning of 180 min of hydration, under environment temperatures of 5 and 20 °C, respectively. In dissolution concentration of calcium ions and sulfate ions of the reference paste, as indicated by Figure 1a and Figure 2e, it can be seen that the initial Ca^2+^ and SO_4_^2−^ concentration reaches 20 mmol/L and 4.2 mol/L at 0–5 min of hydration due to the dissolution of hydrated minerals and gypsum phase in contact with water. Then, during the 0~60 min of hydration, the Ca^2+^ and SO_4_^2−^ concentration decreased slowly due to the continuous generation of ettringite phases and initial C-S-H gel deposited, along with a steady dissolution rate of gypsum phase. Then, the increasing trend of Ca^2+^ concentration is observed from 60 min to 180 min of hydration, corresponding to the lower dissolution rate of alite phases in the later induction period. On the other hand, the SO_4_^2^^−^ concentration continues to decrease slowly in this period, corresponding to the slow formation of ettringite phases and complete dissolved gypsum. Additionally, in this period, sulfate ions are absorbed on the surface of C_3_A phases and the induction period begins. In the measured hydration time, the silicate ions gradually increased from the beginning of 9.4 mmol/L at 5 min to 10.2 mmol/L at 180 min, as shown in Figure 2c.

In the cement paste with AFA-F, lower initial Ca^2+^ dissolution concentrations with 11.1 mmol/L may be determined at hydration of 5 min, associated with the significant consumption of Portlandite and gypsum phases to form the massive ettringite. Furthermore, in this period, the high fluoride content (measured as 34.6 mmol/L) introduced into alkali-free accelerators and the created acid reaction environment promote an exothermic dissolution process of C_3_S phase, thus increasing the silicate ions concentration to the value of 29.1 mmol/L compared with that with the reference paste, which only reaches 9.30 mmol/L. As the hydration continues from 30 min to 180 min, the F^−^ dissolution concentration in this system decreased sharply with a value of 0.90 mmol/L, together with the lower Ca^2+^ leaching concentration, compared with that with AFA-H. This phenomenon may be associated with the precipitation of CaF_2_ crystals from the combination of F^−^ and Ca^2+^.

In the cement paste with AFA-H, except for fluoride ion concentration results, the Ca^2+^ and SO_4_^2−^ concentration curve is very similar to that with AFA-F. Specifically, at 5 min of hydration, the sulfate ions concentration sustains a much higher level observed in cement paste with AFA-H and AFA-F, being approximately 1.5 times than that with reference paste, induced by nearly 50% mass percent of aluminum sulfate octadecahydrate (Al_2_(SO_4_)_3_ 18H_2_O) obtained in the synthesis procedure.

For the hydration at 5 °C, the ions leaching concentration of Ca^2+^, SiO_3_^2−^, SO_4_^2−^, and F^−^ development tendency were almost the same as that at 20 °C. Due to the lower temperature, the leaching concentrations of Ca^2+^ and SO_4_^2−^ are obviously lower than that of the same cement paste system at 20 °C. Nevertheless, the reduction of SiO_3_^2−^ concentration was not that remarkable, along with a dramatically increasing trend of F^−^ concentration. This phenomenon may be related to the lower precipitation effect of CaF_2_ crystals from the combination of F^−^ and Ca^2+^. The lower ion concentration of Ca^2+^, SiO_3_^2−^, and SO_4_^2−^ is responsible for explaining the slower strength development, as shown later in Table 4.

### 3.3. Setting Time and Mortar Strength

The most important performance indicator of setting accelerators is their setting performance. AFA-H and AFA-F can both reach the requirements of setting time which limits the initial setting time and final setting time within 5 min and 12 min, respectively, even under 5 °C conditions. With the existence of fluoride ions, AFA-F presented a better setting acceleration effect even with a lower aluminate sulfate introduction. However, the slow early strength development of AFA-F limits its widespread application in tunneling. It is obvious to see that the compressive strength of mortar with AFA-F cannot reach 1 MPa at 6 h, thus, it was unable to play an effective support role at the early age when the tunnel deformation is still developing. Additionally, the 12 h, 24 h, and 48 h compressive strength of mortars with AFA-F was 45.1%, 58.6%, and 74.1% of the mortars with AFA-H, respectively, indicating that fluoride ions obtained in accelerators reduce the rate of early strength development of Portland cement systems. In addition to this, the development of compressive strength of the mortars with AFA-F from 12 h to 72 h was all lower than that of the reference mortars. In addition, the negative impact of the 5 °C temperature on strength development became less and less as the environment age increased for the mortars with alkali-free setting accelerators. To be specific, the 6 h compressive strength of mortars with AFA-H at 5 °C was 27.8% of that at 20 °C, while the proportion went to 45.1%, 58.6%, 74.1%, and 92.4% at the age of 12 h, 24 h, 48 h, and 72 h, respectively.

### 3.4. X-ray Diffraction Analysis

**Figure 3:**
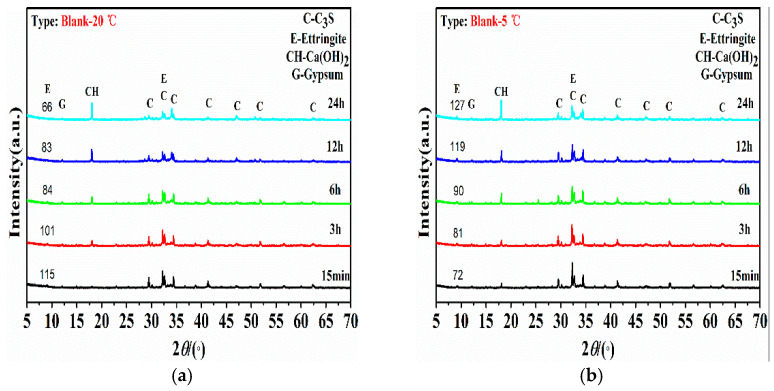

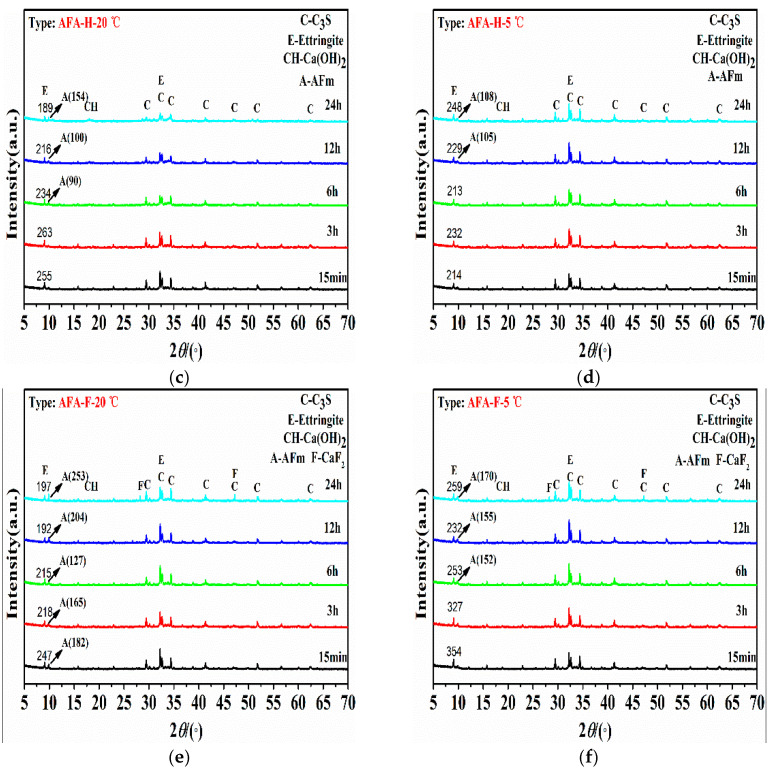
Evolution of XRD patterns in the cement paste with reference (**a**,**b**), non-fluorine alkali-free setting accelerators (**c**,**d**), and fluorine-containing alkali-free setting accelerators (**e**,**f**) during the first 24 h of hydration under 20 °C or 5 °C environment temperatures. (**a**) XRD patterns of the reference paste^-^ at 20 °C, (**b**) XRD patterns of the reference paste at 5 °C, (**c**) XRD patterns of the AFA-H paste^-^ at 20 °C, (**d**) XRD patterns of the AFA-H paste at 5 °C, (**e**) XRD patterns of the AFA-F paste^-^ at 20 °C, (**f**) XRD patterns of the AFA-F paste at 5 °C.

Figure 3 shows the effect of liquid alkali-free setting accelerators on the early hydrate phase of Portland cement under 20 °C or 5 °C environment temperature. In Figure 3a,b, the characteristic peaks of hydration products of reference cement paste samples after hydration for 15 min, 3 h, 6 h, 12 h, and 24 h are analyzed by XRD. As seen in Figure 3a,b, the early hydride products of the reference cement paste system were dominated by unhydrated C_3_S, ettringite, and Ca(OH)_2_. No more C_3_A components were found in these figures, which may be covered by the C-S-H gel hydrates. In particular, the amount of hydration product of Ca(OH)_2_ is proportional to the hydration age, with the most rapid increase in 6–12 h, which is consistent with the exothermic behavior of hydration when Portland cement is in the accelerated hydration period. The peak intensity of ettringite phases weakened slowly from 15 min to 24 h, which may be related to its later hydration to transform into monosulfoaluminate hydrate (AFm), while the absence of AFm phase in the XRD results may be related to its low content and weak crystallization form. But in reference paste under 5 °C environment temperature, the peak intensity of ettringite increased slowly from 15 min to 24 h, which indicates that the initial hydration process of C_3_A phases is significantly retarded and the consumption of ettringite into AFm is also restrained at the lower temperature. Meanwhile, the peak intensity of ettringite for the reference cement paste was all below the level of accelerated cement pastes with alkali-free accelerators. Figure 3c,d represent the XRD analysis of cement pastes with AFA-H under environment temperature of 20 °C or 5 °C. At 15 min of hydration, a significantly increased content of hydration products ettringite is obviously observed in accelerated cement paste, but no hydration products, such as Ca(OH)_2_ produced by C_3_S hydration, are found, indicating that the rapid setting effect of alkali-free setting accelerators is related to the rapid nucleation growth of ettringite, and the process is accompanied by the continuous consumption of Ca(OH)_2_, resulting in a hydration environment in which Ca(OH)_2_ had not reached the level of saturation precipitation. At the hydration time of 12~24 h in the 20 °C environment condition, Ca(OH)_2_ was accelerated by C_3_S hydration and started to nucleate and crystallize, leading to the weaker C_3_S peak intensity. Meanwhile, the mortar strength developed rapidly during the same period, indicating that the early strength development of the accelerated cement system is closely related to the further hydration of C_3_S.

Figure 3c shows that the relative peak intensity of ettringite in the cement paste with AFA-H weakened just after 3 h of hydration, indicating that the rapid setting action produced a higher amount of ettringite phases, which provided a larger growth base for the nucleation rate of AFm. It can be clearly seen that the peak intensity of AFm is at the hydration time of 6~24 h in Figure 3c. As for the 5 °C environment condition, even the ettringite generated in the beginning of the hydration at the 20 °C, the C_3_S peak intensity was not significantly decreased and the peak intensity of AFm can be seen only from the hydration time of 12 h. Thus, the strength development was notably slowed at the low temperature which is represented in Table 4.

**Figure 4:**
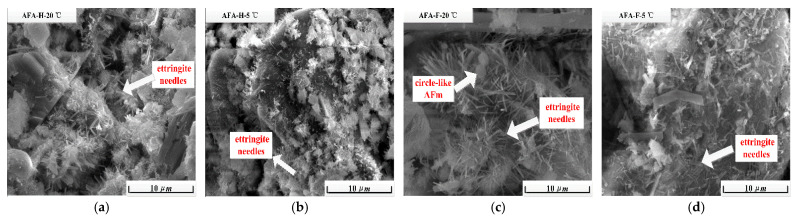
At 15 min of hydration, ESEM images of cement paste containing alkali-free accelerators without (**a**,**b**) and with fluoride (**c**,**d**) under 20 °C or 5 °C environment temperatures.

As seen in Figure 4c, the peak intensity of ettringite formed in the cement paste with AFA-F after 15 min of hydration decreased, probably because F^−^ formed a relatively stable fluoro-aluminum complex structure with Al^3+^ generated by the dissolution of C_3_A, thus inhibiting the formation of [20], which in turn delayed the nucleation process of calcium alumina by weakening the formation process of aluminum-oxygen octahedra [21]. This indicates that the enhancement of the quick-setting effect of fluoride on the alkali-free setting accelerator was not related to the quantity of ettringite formed. The AFm products have been identified first at the hydration of 15 min in Figure 3e, decreased until 6 h, and then increased with the extension of hydration time, indicating the ettringite transforming rate is quite rapid and early. Thus, with more ettringite generated along with the hydration, the peak intensity of AFm weakened. However, for cement paste with AFA-F at 5 °C, the peak intensity of AFm can be recognized at the hydration of 6 h and the peak intensity of ettringite phases was higher than that of the cement paste at 20 °C, causing a lower strength development of the mortar specimen. After 24 h of hydration, the amount of Ca(OH)_2_ formed by the accelerated hydration of C_3_S in the cement pastes with setting accelerators was significantly less than that in the reference cement paste, but the consumption of C_3_S was obviously much less for cement paste with AFA-F at 5 °C and 20 °C environment condition and accompanied by the precipitation of the new product crystalline calcium fluoride (CaF_2_), leading to lower strength development. Combined with the results of the decrease in cement hardening rate of the mortars with AFA-F, it indicates that during the accelerated hydration period, the F^−^ of the system participated in the C3S hydration process and generated CaF_2_ products with Ca^2+^, which has adsorption and blocking effects on C_3_S or C-S-H gels, increasing the migration steric hindrance of liquid phase ions to the hydrated mineral surface, thus slowing down its hydration process.

### 3.5. ESEM Microanalysis

ESEM images obtained in the accelerated cement paste at 15 min of hydration under 20 °C or 5 °C are shown in Figure 4a–d. In the cement paste with non-fluorine alkali-free accelerators under 20 °C (Figure 4a), massive needle-like ettringite and fewer Portlandite phases are easily recognized, as observed in Figure 3b, while the content and length of such ettringite phases may be reduced by the lower environment temperatures from 20 °C to 5 °C, as shown in Figure 4b. Once the fluorine compound is added into alkali-free accelerators, the quick setting hydration products are mainly comprised of needle-like ettringite [22] and plate-like monosulfoaluminate phases. Similarly, this quick setting hydration process is weakened at 5 °C with shorter needle-like ettringite phases and the content of monosulfoaluminate phases is reduced obviously to an unmeasurable amount, which is unable to be detected by the XRD instrument.

**Figure 5:**
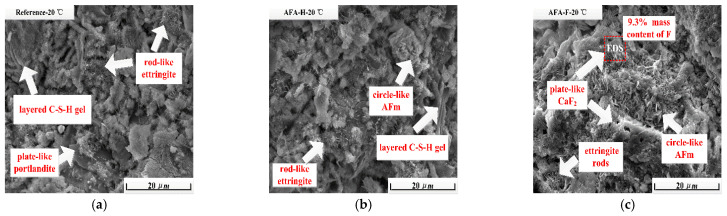
At 20 °C environment temperature, ESEM images of cement paste with reference one (**a**), alkali-free accelerators without fluoride (**b**), and with fluoride (**c**) at 24 h of hydration.

ESEM images obtained in cement paste at 24 h of hydration under 20 °C are shown in Figure 5a–c. In the reference cement paste (Figure 5a), rod-like ettringite phases and layered C-S-H gels together with Portlandite phases are found, supporting the accelerating hydration stages of alite minerals. Nevertheless, it is still difficult to detect the Portlandite phases in accelerated cement paste. In the cement paste with non-fluorine alkali-free accelerators, the needle-like ettringite phase at 15 min of hydration tends to be rod-like and converts into circle-like AFm phases at 24 h of hydration. With fluorine-containing alkali-free setting accelerators introduced, plate-like CaF_2_ together with circle-like AFm phases are observed, and possibly intercalate with similar layered C-S-H gel hydrates and are deposited on cement grains [23], as detected by the XRD instrument in Figure 3c.

**Figure 6:**
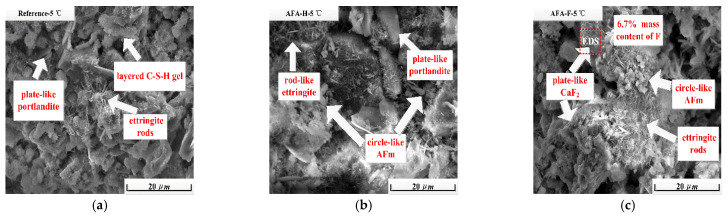
At 5 °C environment temperature, ESEM images of cement paste with reference one (**a**), alkali-free accelerators without fluoride (**b**), and with fluoride (**c**) at 24 h of hydration.

ESEM images obtained in cement paste at 24 h of hydration under 5 °C are shown in Figure 6a–c. As indicated by Figure 6a, once the environment temperature is decreased from 20 °C to 5 °C, thinner rod-like ettringite phases and less compact layered C-S-H gels are observed in the reference cement paste, which supports the delayed hydration effect induced by low environment temperature. In the accelerated cement paste, the content of layered C-S-H gels is reduced obviously, which implies the delayed hydration process of alite minerals and lower initial compressive strength. Meanwhile, the transformation rate from ettringite phases to AFm phases is slowed down at low environment temperatures, behaving less content of AFm phases. Specifically, the existence of CaF_2_ phases and its negative effect on C-S-H gel are proved again with the ESEM images obtained from the cement paste with fluorine-containing alkali-free setting accelerators under 5 °C.

## 4. Conclusions

This study mainly evidences how early age hydration and setting and hardening characteristics of a type of reference cement are affected by low environment temperatures in the presence of different alkali-free accelerators. The results obtained from physico-chemical experimental tests support the performance difference of wet-mix shotcrete accelerated by alkali-free accelerators with or without fluoride compounds, such as the ability to shorten setting times and early age hydration behavior. The following conclusions can be drawn based on the analysis results using different experimental methods.

(1) Compared with that with the non-fluoride alkali-free accelerators, alkali-free accelerators composed of aluminum sulfate and fluoride compounds promote the alite minerals dissolution and more calcium and silica ions are released in the liquid phase of the cement paste. Depending on the sufficient sulfate obtained in the setting accelerators synthesis procedure, more highly exothermic ettringite and monosulfoaluminate hydrate form quickly, hence leading to a relatively shorter setting time and coagulation effect.
(2) The early age hydration process of accelerated cement pastes is delayed when the environment temperature decreased from 20 °C to 5 °C, causing a longer setting time and lower initial mortar strength. Nevertheless, in the cement system with fluorine-containing alkali-free setting accelerators, its setting performance seems to be less sensitive to the negative initial hydration effect induced by a low temperature of 5 °C, compared with that without fluorides. This phenomenon may be associated with the fact that the promoted dissolution effect of alite minerals was not disturbed and silicate ions concentration was still as high as that at 20 °C. Furthermore, quick setting hydration products like ettringite phase content were not reduced from the XRD results.
(3) The initial mechanical strength of accelerated cement mortar is lower with fluorine compounds introduced into alkali-free setting accelerators. As measured by isothermal calorimetry and XRD analysis, fluorine ions are easily combined with calcium ions to form calcium fluoride products in the early hydration stage. It is observed that calcium fluoride products, which have a thin-plate shape and low structural strength, tend to intercalate with similar layered C-S-H gel hydrates and weaken the denseness of hydration products. The existence of fluorine ions promotes the conversion of ettringite to monosulfoaluminate hydrate in the accelerating period, which blocks alite dissolution sites and inhibits its further hydration, resulting in slower strength development.

## Figures and Tables

**Table 1:** Chemical and mineralogical compositions of the cement.

Chemical Composition (%)		Mineralogical Composition (%) ^1^	
Compound	Content	Compound	Content
LOI	5.64	C_3_S	55.0
SiO_2_	18.379	C_2_S	11.2
Al_2_O_3_	4.407	C_3_A	4.7
CaO	61.956	C_4_AF	12.5
Fe_2_O_3_	3.244	CaSO_4_	1.2
MgO	2.434	Ca(OH)_2_	2.8
Na_2_O	0.121	CaCO_3_	0.8
K_2_O	0.594	MgO	1.1
SO_3_	2.880	SiO_2_	0.2
TiO_2_	0.343	K_2_SO_4_	0.7
Total sum	99.98	CaSO_4_·2H_2_O	0.6
		CaSO_4_·1/2H_2_O	2.0
		Dolomite	1.2
		Amorphous	5.9
		Total sum	99.9

^1^ semi-quantitatively analyzed by Rietveld analysis method.

**Table 2:** Physical properties and chemical compositions of AFA.

Material	Unit	AFA-F	AFA-H
Density	g/cm^3^	1.39	1.45
Solid content	%	49.5	61.3
pH	/	2.4	3.1
Stability	mL	3	1
Chloride Content	%	0	0
Fluoride content	%	2.7	0

**Table 3:** Cement paste and mortar compositions for each accelerator and environment temperature.

Properties	Reference Paste	AFA-H Paste	AFA-F Paste	Reference Mortar	AFA-H Mortar	AFA-F Mortar
Cement (g)	400.0	400.0	400.0	900.0	900.0	900.0
Deionized water	140.0	123.8	127.6	450.0	413.6	422.1
GSB 08-1337 sand	/	/	/	1350.0	1350.0	1350.0
AFA-H (% bcw)	0	32	32	0	72	72
AFA-F (% bcw)	0	32	32	0	72	72

**Table 4:** Setting time of accelerated cement paste and mortar compressive strength at early age.

Group	Dosage /%	Measured Temperature/°C	Setting Time/min		Compressive Strength/MPa				
			Initial	Final	6 h	12 h	24 h	48 h	72 h
Ref.	/	20	205 ^1^	315 ^1^	/	1.5	13.5	25.6	35.7
		5	258 ^1^	425 ^1^	/	0.6	8.5	13.6	23.5
AFA-H	8	20	2:10	5:40	1.8	5.1	17.4	22.4	29.1
	8	5	3:30	8:00	0.5	2.3	10.2	16.6	26.9
AFA-F	8	20	1:00	1:20	0.1	0.3	6.9	16.5	19.1
	8	5	1:20	2:00	/	/	3.8	10.8	16.8

^1^ measured with Chinese standard GB/T 1346-2011.

## Data Availability

The data presented in this study are available on request from the corresponding author. The data are not publicly available due to a series of studies were conducted by the product team.
